# Treatment interventions for diarrhoea in HIV-infected and HIV-exposed children: a systematic review

**DOI:** 10.11604/pamj.2018.29.208.15240

**Published:** 2018-04-09

**Authors:** Nkengafac Villyen Motaze, Chukwuemeka Nwachukwu, Eliza Humphreys

**Affiliations:** 1 Centre for Development of Best Practices in Health (CDBPH), Yaoundé Central Hospital, Yaoundé, Cameroon; 2 Cochrane South African, South African Medical Research Council, Cape Town, South Africa; 3 Centre for Vaccines and Immunology, National Institute for Communicable Diseases, South Africa; 4 Department of Global Health, Faculty of Medicine and Health Sciences, Stellenbosch University, South Africa; 5 Excellence & Friends Management Consult (EFMC), Abuja, Nigeria; 6 Global Health Sciences, University of California, San Francisco, San Francisco, California, USA

**Keywords:** Diarrhoea, human immunodeficiency virus, children

## Abstract

**Introduction:**

Seventy percent of an estimated 10 million children less than five years of age in developing countries die each year of acute respiratory infections, diarrhoea, measles, malaria, malnutrition or a combination of these conditions. Children living with Human immunodeficiency virus (HIV) are at risk of diarrhoea because of drug interactions with antiretroviral therapy and bottle feeding. This may be aggravated by malnutrition and other infectious diseases which are frequent in children living with HIV. Objective: to evaluate treatment interventions for diarrhoea in HIV infected and exposed children.

**Methods:**

A comprehensive search was conducted on 02 June 2016 to identify relevant studies for inclusion. We included randomised controlled trials of HIV infected or exposed children under 15 years of age with diarrhoea. Two authors independently selected studies for inclusion, assessed risk of bias (RoB) and extracted data using a pre-designed data extraction form.

**Results:**

We included two studies (Amadi 2002 and Mda 2010) that each enrolled 50 participants. The RoB was assessed as low-risk for both included studies. There was no difference in clinical cure and all-cause mortality between nitazoxanide and placebo for cryptosporidial diarrhoea in Amadi 2002. In Mda 2010, there was a reduction in duration of hospitalisation in the micronutrient supplement group (P < 0.005) although there was no difference in all-cause mortality.

**Conclusion:**

There is low certainty evidence on the effectiveness of nitazoxanide for treating cryptosporidial diarrhoea and micronutrient supplementation in children with diarrhoea. Adequately powered trials are needed to assess micronutrients and nitazoxanide, as well as other interventions, for diarrhoea in HIV-infected and-exposed children.

## Introduction

It is estimated that over 10 million children in developing countries die each year before they reach five years of age. Seventy percent of these deaths are due to acute respiratory infections (mostly pneumonia), diarrhoea, measles, malaria, or malnutrition or a combination of these conditions [1]. Diarrhoeal diseases alone account for an estimated 17.5-21% (about 2.5 million) of all deaths in children in this age range [2, 3]. This high disease burden persists despite effective prevention strategies like hand washing [4], improved disposal of human feaces [5] and improved quality of water [6]. According to the Joint United Nations Program on human immunodeficiency virus (HIV) and acquired immune deficiency syndrome, (AIDS) 33.3 million people were living with HIV worldwide at the end of 2009 and 2.5 million were children [7]. Children acquire HIV from their mothers at three distinct periods; during intra-uterine life (when the child is in the womb), during delivery and during breastfeeding. In addition to these, other situations in which HIV transmission can occur, though less frequently, are nosocomial transmission (infection during hospital care) and sexual abuse. In 2009, an estimated 370 000 children contracted HIV during the perinatal and breastfeeding period [7]. The risk of transmission during breastfeeding alone is about 10%-16% while intrauterine and perinatal (during labour and delivery) transmission account for 25-40% and 60-75% of infections, respectively [8]. However, there has been pronounced progress in reducing the incidence and impact of HIV among children younger than 15 years due to progress in prevention strategies like prevention of mother to child transmission (PMTCT) programs through the use of antiretroviral (ARV) drugs. A reflection of these efforts is that 32% fewer children were newly infected and 26% fewer deaths occurred from AIDS-related conditions among children in 2009 compared with 2004 [7]. Children who are considered HIV-exposed include infants who are born to HIV infected mothers and/or breastfeeding from an HIV infected mother. Infection should have been excluded in those born to HIV infected mothers (did not acquire HIV during intra-uterine life or delivery) and in those who were breastfeeding from an HIV infected mother after cessation of breast milk. These children are at higher risk of mortality compared to children who are not exposed to HIV [9, 10]. Findings from observational studies estimate that the risk of transmission of the HIV virus to the child due to breastfeeding is 14% [11]. PMTCT guidelines recommend that children receive anti-retroviral therapy (ART) during the entire breastfeeding period, in order to reduce HIV transmission [12]. Exclusive breastfeeding has been found to be associated with a reduced risk of HIV transmission compared to mixed feeding [13]. While an estimated 1.7 million babies have been infected with the HIV virus through breast milk, up to 1.5 million babies die every year because they are not breast fed [14]. Some children who are born to HIV positive mothers are fed with formula. If safe water and adequate sanitation are not available, there is an increased risk of diarrhoeal disease from formula feeding. An estimated 88% of diarrhoeal deaths worldwide are attributable to unsafe water, inadequate sanitation and poor hygiene [15]. Therefore, the risk of transmission through breastfeeding has to be weighed against the risk of diarrhoeal diseases when choosing a method of feeding in HIV infected mothers. Treatment of diarrhoea involves fluid replacement, reducing intestinal motility, targeting the causative organism and replenishing micronutrients. The basis of rehydration during diarrhoea is to replace body water that is lost through frequent emission of loose stools. Since electrolytes are also lost along with water, several types of glucose-based ORS with different compositions and osmolarities are available that can compensate this loss.

Consumption of fluids that have high osmolarities can lead to secretion of water into the intestines and aggravation of dehydration. Decreasing the osmolarity of the ORS solution to 245 mOsm/l by reducing the concentrations of glucose and salt avoids this adverse effect and improves the efficacy of the ORS regimen for children with acute non-cholera diarrhoea [16]. A new type of ORS, which is a polymer-based ORS, has been shown to decrease the duration of diarrhoea among adults with cholera and lower the risk of unscheduled use of intravenous fluid, compared with a glucose-based ORS >310 mOsm/l [17]. Antibiotics for treating specific opportunistic infections that cause diarrhoea [8], target the causative germs and eradicate them in order to stop the diarrhoeal episode. However, this has to be done concurrently with adequate rehydration and feeding for optimal recovery. Replacing depleted micronutrients (such as Zinc) has a preventive and long-lasting impact by reducing the number of episodes of diarrhoea in the 2-3 months after the supplementation regimen [16]. Other treatment interventions like anti motility agents have been shown to benefit children with acute diarrhoea [18]. A recent systematic review found that probiotics administered in addition to rehydration therapy resulted in significant reductions in the duration and severity of acute infectious diarrhoea [19], however another review found no benefit in persistent diarrhoea [20]. In addition to the above management strategies, there are additional considerations for children living with HIV in whom there is a potential for drug interactions with antiretroviral therapy, the possibility of exclusive bottle feeding (hence less breast milk) and the wider variety of pathological agents. This may be aggravated by malnutrition and other infectious diseases which are frequent in children living with HIV. Recent Cochrane reviews confirm the benefit of zinc supplementation [21], rehydration via oral hydration solution or intravenous solutions and antibiotics for bloody diarrhoea [22-24]. However, these reviews do not address subpopulations of HIV infected or exposed children, who may be especially vulnerable to diarrhoeal disease. Infants and children with HIV/AIDS have diarrhoeal disease complicated by immunocompromise, malnutrition, gastrointestinal manifestations of primary HIV disease, and may be treated with ARV drugs, that are associated with gastrointestinal symptoms, among other challenges [25-27]. The combination of HIV infection and diarrhoea thus represents a complex and challenging situation that is faced by health workers worldwide and especially in developing countries. WHO has set research on childhood diarrhoea as a priority area in order to meet the United Nation's Millennium Development goal of reducing childhood mortality by two-thirds between 1990 and 2015 [28]. Also recognizing that children with HIV require comprehensive care, WHO has initiated a series of systematic reviews that will underlie evidence-based recommendations on the prevention and treatment of common conditions in HIV-infected and exposed children, including diarrhoea [29]. In this review, we intend to summarise the evidence and outcomes for the recommended treatment interventions for diarrhoea in infants and children with HIV infection and exposure.

## Methods

Details of the methodology used in this systematic review have been previously published [30]. In summary, we searched for and included randomised controlled trials (RCTs) that enrolled children less than 15 years of age who are HIV infected or exposed and who have diarrhoea (reported by the carer and confirmed by the health professional). HIV-exposed infants are defined as infants born to HIV infected mothers and/or breastfeeding from an HIV infected mother and in whom infection has been excluded after birth in those born to HIV infected mothers (did not acquire HIV during intra-uterine life or delivery) and after cessation of breast milk in those who were breastfeeding from an HIV infected mother. Children who are HIV-infected were those in whom a laboratory diagnosis was made. The primary outcomes were clinical cure (defined as cessation of diarrhoeal episodes) and all-cause mortality. Secondary outcomes included: hospitalisation due to diarrhoea; Severity of diarrhoea (stool output: consistency and frequency); mortality due to dehydration from diarrhoea; adverse treatment events; and recurrence of diarrhoea. We formulated a comprehensive search strategy with the assistance of the HIV/AIDS Review Group Trials Search co-coordinator, in order to identify all relevant published and unpublished studies. The search period was from 1 January 1980 to the 2 of June 2016 and included studies conducted in all of countries irrespective of language of publication. The first two review authors independently assessed identified studies for inclusion, extracted data and conducted risk of bias assessment using the criteria outlined in the Cochrane Handbook for Systematic Reviews of Interventions [31]. We resolved any disagreement through discussion and if required, we consulted the third author for a final decision. Study data was entered into review manager software [32] for analysis. We assessed the certainty of evidence using the Grading of Recommendations Assessment, Development and Evaluation (GRADE) approach [33]. We defined the certainty of evidence for each outcome as the extent to which one can be confident that an estimate of effect or association is close to the quantity of specific interest [31]. The rating across studies has four levels: high, moderate, low or very low. Randomised controlled trials are categorised as high certainty but can be downgraded; similarly, other types of controlled trials and observational studies are categorised as low quality but can be upgraded [33].

## Results

**Figure 1:**
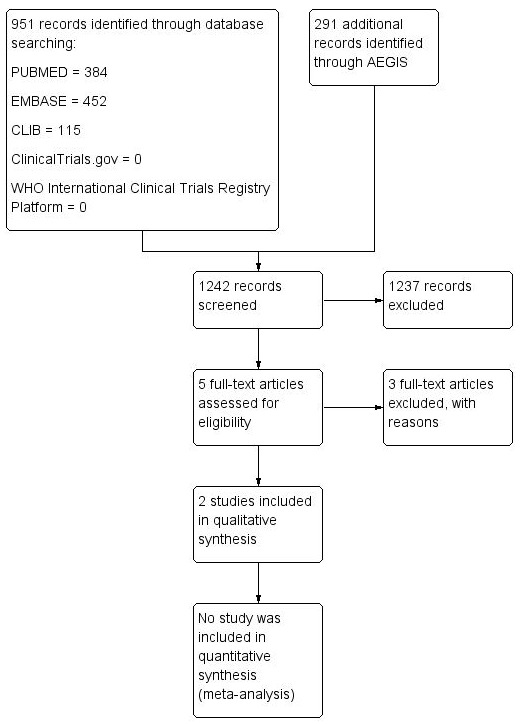
Study flow diagram showing study selection and data extraction process at each stage of the review

**Figure 2:**
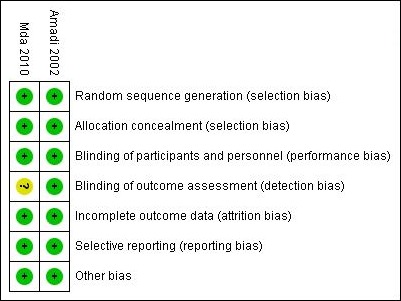
Risk of bias summary for included studies according to the Cochrane risk of bias domains for randomised controlled trials

The results of the search (02 June 2016) are summarised in the PRISMA flow diagram (Figure 1). There were 1242 records identified using the search strategy. Of these, five were identified as being potentially relevant and two randomised controlled trials (Table 1) were included following assessment of full text articles. Overall, these two studies had a low risk of bias according to the Cochrane risk of bias tool (Figure 2). In South Africa, Mda 2010 [34] compared micronutrient supplementation to placebo for treating HIV-infected children with diarrhoea who were admitted to the paediatric ward of the Dr George Mukhari hospital which is a tertiary referral institution. Fifty HIV-infected children admitted with diarrhoea and aged 4 months to 2 years, were randomised; 24 to intervention group and 26 to control group (1 participant in the control group was excluded following randomisation). Children who had diarrhoea lasting above 72 hours, who had received any micronutrient, had any chronic illnesses, or were on antiretroviral treatment, were not eligible for inclusion. The second trial, Amadi 2002 [35], was conducted in Zambia. It included children above one year of age with cryptosporidial diarrhoea, whose parents agreed to perform an HIV test. The trial was conducted between November 2000 and July 2001 at the Department of Paediatrics and Child Health, University Teaching Hospital, or to any of four urban health centres managed by the Lusaka Urban District Health Management Board. Fifty HIV-infected children were randomised in a 1:1 ratio to either nitazoxanide or placebo. Children who had received any antiprotozoal drug within two weeks of enrolment were not included in the study. Three studies were excluded from this review. Two studies; Amadi 2005 [36] and Luabeya 2007 [37] because of ineligible participants and one study, Rollins 2007 [38] because of ineligible outcomes.

**Table 1:** Risk of bias assessment for included studies

Amadi 2002		
Risk of Bias domain	Authors’ judgement	Support for judgement
Random sequence generation	Low risk	Computer-generated random numbers
Allocation concealment	Low risk	The drug and placebo were supplied in pre-coded containers according to the randomisation code.
Blinding of participants and personnel	Low risk	Identical formulation of drug and placebo was used.
Blinding of outcome assessors	Low risk	Outcome assessors were not aware of treatment assignment.
Incomplete outcome data	Low risk	All randomised participants accounted for.
Selective reporting	Low risk	All relevant outcomes were reported
Other bias	Low risk	No other source of bias identified.
**Mda 2010**		
Risk of Bias domain	Authors’ judgement	Support for judgement
Random sequence generation	Low risk	Computer-generated random numbers
Allocation concealment	Low risk	Identical containers labelled by study serial number
Blinding of participants and personnel	Low risk	Identical appearance and taste of supplement and placebo tablets.
Blinding of outcome assessors	Unclear risk	No detailed information provided on blinding of outcome assessors
Incomplete outcome data	Low risk	No loss to follow-up
Selective reporting	Low risk	All relevant outcomes were reported
Other bias	Low risk	No other source of bias identified.

## Effects of interventions

**Anti-infective agents:**Amadi 2002 was conducted in Zambia and compared 5ml (100mg) of 20g/l nitazoxanide oral suspension given twice daily for three days to an identical placebo in HIV-infected children with diarrhoea due to cryptosporidiosis. There was no difference (P value = 1.0) in all-cause mortality between the nitazoxanide group 5/25 (20%) and the placebo group 4/24 (17%). There were 2/25 (8.0%) children in the nitazoxanide group assessed as being clinically cured at day 7 of hospitalisation compared to 6/24 (25.0%) in the placebo group (P = 0.14).

**Micronutrient supplementation:**The effect of short-term micronutrient supplementation on diarrhoea was assessed in Mda 2010. A micronutrient supplement (containing: retinol (300µg), thiamine (0.6mg), Riboflavin (o.6mg), Niacin 8mg), Pyridoxine (0.6mg), Cobalamine (1 µg), Folic Acid (70 µg), Ascorbic Acid (25mg), 1,25 dihydro cholecalciferol (5 µg), α-Tocopherol (7 mg), Copper (700 µg), Iron (8mg), Selenium (30 µg) and Zinc (8mg)) was compared to an identical placebo and administered as crushable tablets once daily for the entire duration of hospitalisation. All-cause mortality did not differ between study arms with 2/24 (8.3%) in the micronutrient supplement group and 2/26 (7.7%) in the placebo group. The duration of hospitalisation in the micronutrient group (mean (SD) = 7.0 days (4.5)) was shorter (P < 0.005) than the placebo group (mean(SD) = 8.6 days (5.0)). None of the included studies reported on adverse effects of the interventions.

## Discussion

There were no summary effect estimates in this review and the results of the studies are provided in a narrative manner. The two included trials each investigated different interventions compared to placebo in HIV-infected children with diarrhoea. In Amadi 2002, nitazoxanide did not reduce all-cause mortality and proportion of children clinically cured in HIV-infected children with cryptosporidial diarrhoea. On the other hand, there was a reduction in duration of hospitalisation in the micronutrient supplement group (P < 0.005) in Mda 2010 although there was no difference in all-cause mortality. The Grading of Recommendations Assessment, Development and Evaluation (GRADE) [33] approach was used to assess the quality of evidence in this review. Two comparisons were made, each addressed by one included study; nitazoxanide versus placebo for Mda 2010 (Table 2) and micronutrients versus placebo for Amadi 2002 (Table 3). Both included studies were randomised controlled trials so we initially qualified the evidence as ´very high´ before downgrading by two points for serious imprecision due to the very small sample sizes. We therefore concluded that there was low-certainty evidence that micronutrients are more effective compared to placebo for treating HIV-infected children with diarrhoea. We also concluded that there was low-certainty evidence in favour of nitazoxanide compared to placebo for treating HIV-infected children with cryptosporidial diarrhoea. It is likely that these results are due to small sample sizes in the included studies, rendering them underpowered to detect differences in outcomes between intervention groups. If further trials are carried out enabling a larger number of participants and events in the study, the estimates of intervention effects and the certainty of evidence will likely be different. With respect to the generalisability of the results, included studies enrolled male and female HIV-infected children in Zambia and South Africa. The ages ranged from 4 to 24 months in Mda 2010 and 12 to 85 months in Amadi 2002. The race, level of education and socio-economic status of the participants were not reported. Black Africans are the most common racial group while income levels and maternal education could vary significantly among individuals in these countries. The findings of this review therefore apply to the HIV-infected African children and the effects of the interventions might differ in other population subgroups.

**Table 2:** Summary of findings table for Nitazoxanide versus placebo in HIV-infected and -exposed children with cryptosporidial diarrhoea

**Patient or population:** HIV-infected and HIV-exposed children with cryptosporidial diarrhoea **Settings:** Tertiary hospital in Zambia **Intervention:** Nitazoxanide versus placebo						
**Outcomes**	**Illustrative comparative risks^+^ (95% CI)**		**Relative effect (95% CI)**	**No of Participants (studies)**	**Certainty of the evidence (GRADE)**	**Comments**
	Assumed risk	Corresponding risk				
	Placebo	Nitazoxanide				
**Clinical cure** Last unformed stool assessed by clinician Follow-up: 7 days	See comment	See comment	Not estimable	50 (1 study)	⊕⊕⊝⊝ **low**^1^	N/A
**All-cause mortality** Assessed by hospital personnel Follow-up: 8 days	See comment	See comment	Not estimable	50 (1 study)	⊕⊕⊝⊝ **low**^1^	N/A

+ The basis for the assumed risk is the control group risk across the included studies. The corresponding risk (and its 95% confidence interval) is based on the assumed risk in the comparison group and the relative effect of the intervention (and its 95% CI).

1 Downgraded by two points for imprecision: only one small study reported on this outcome

GRADE Working Group grades of evidence

**High quality:** Further research is very unlikely to change our confidence in the estimate of effect. **Moderate quality:** Further research is likely to have an important impact on our confidence in the estimate of effect and may change the estimate. **Low quality:** Further research is very likely to have an important impact on our confidence in the estimate of effect and is likely to change the estimate. **Very low quality:** We are very uncertain about the estimate

**Table 3:** Summary of findings table for micronutrients versus placebo in HIV-infected and -exposed children with diarrhoea

**Patient or population:** HIV-infected and HIV-exposed children with diarrhoea **Settings:** Tertiary referral centre in a peri-urban area of South Africa **Intervention:** Micronutrients versus placebo						
**Outcomes**	**Illustrative comparative risks^+^ (95% CI)**		**Relative effect (95% CI)**	**No of Participants (studies)**	**Certainty of the evidence (GRADE)**	**Comments**
	**Assumed risk**	**Corresponding risk**				
	**Placebo**	**Micronutrients**				
**Clinical cure**	See comment	See comment	Not estimable	N/A	See comment	Not measured
**All-cause mortality**: Assessed by clinician at study site	See comment	See comment	Not estimable	50 (1 study)	⊕⊕⊝⊝ **low**^1^	N/A
**Hospitalisation due to diarrhoea:** Measured by medical staff as time to cessation of diarrhoea	See comment	See comment	Not estimable	44 (1 study)	⊕⊕⊝⊝ **low**^1^	N/A

+ The basis for the assumed risk is the control group risk across the included studies. The corresponding risk (and its 95% confidence interval) is based on the assumed risk in the comparison group and the relative effect of the intervention (and its 95% CI).

1 Downgraded by two points for imprecision: only one small study reported on this outcome

GRADE Working Group grades of evidence

**High quality:** Further research is very unlikely to change our confidence in the estimate of effect.

**Moderate quality:** Further research is likely to have an important impact on our confidence in the estimate of effect and may change the estimate

**Low quality:** Further research is very likely to have an important impact on our confidence in the estimate of effect and is likely to change the estimate

**Very low quality:** We are very uncertain about the estimate

## Conclusion

The low-certainty of evidence from this review implies that health care workers treating HIV infected children who have diarrhoea should weigh the benefits against the risks when making a decision on the use of nitazoxanide or micronutrients. There is a need for adequately powered trials assessing micronutrients, nitazoxanide and other interventions (e.g. oral rehydration therapy, anti-diarrhoeal agents and other specific anti-infective agents) for diarrhoea in HIV -infected and -exposed children. Furthermore, trials in children on ART addressing diarrhoea caused by ART-related drug interactions are required since these challenges are specific to this population. The two included studies were carried out in tertiary hospitals located in South Africa and Zambia which are lower middle income countries with a predominantly Black-African population. There is therefore a need for trials conducted in other countries or continents with participants of different races and socio-economic contexts.

### What is known about this topic

- Treatment with antiretroviral therapy reduces opportunistic infections including those that cause diarrhea;
- Symptomatic treatment of diarrhea with oral rehydration therapy and antidiarrhoeal agents can improve disease outcome.

### What this study adds

- This systematic review conducted following Cochrane methodology found that nitazoxanide may make little or no difference in HIV-infected children with cryptosporidial diarrhea and that micronutrient have little or no effect on HIV-infected children with diarrhea;
- We found no clinical trials on treatment of diarrhea in HIV-exposed children so there is a need for further clinical trials in this sub-population.

## Competing interests

The authors declare no competing interests.

## References

[1] Weltgesundheitsorganisation (2005). Handbook IMCI: integrated management of childhood illness.

[2] Kosek M, Bern C, Guerrant RL (2003). The global burden of diarrhoeal disease, as estimated from studies published between 1992 and 2000. Bull World Health Organ.

[3] Boschi-Pinto C (2008). Estimating child mortality due to diarrhoea in developing countries. Bull World Health Organ.

[4] Ejemot-Nwadiaro RI, Ehiri JE, Meremikwu MM, Critchley JA (2008). Hand washing for preventing diarrhoea In: the Cochrane Collaboration, editor Cochrane Database of Systematic Reviews.

[5] Clasen TF, Bostoen K, Schmidt WP, Boisson S, Fung IC-H, Jenkins MW (2010). Interventions to improve disposal of human excreta for preventing diarrhoea. Cochrane Database Syst Rev.

[6] Clasen TF, Alexander KT, Sinclair D, Boisson S, Peletz R, Chang HH (2015). Interventions to improve water quality for preventing diarrhoea. Cochrane Database Syst Rev.

[7] Swiatowa Organizacja Zdrowia (2010). Global report: UNAIDS report on the global AIDS epidemic 2010.

[8] Bartlett JG, Gallant JE, Conradie FM (2008). Medical Management of HIV Infection-South African edition.

[9] Marinda E, Humphrey JH, Iliff PJ, Mutasa K, Nathoo KJ, Piwoz EG (2007). Child mortality according to maternal and infant HIV status in Zimbabwe. Pediatr Infect Dis.

[10] Shapiro RL, Lockman S, Kim S, Smeaton L, Rahkola JT, Thior I (2007). Infant Morbidity, Mortality and Breast Milk Immunologic Profiles among Breast-Feeding HIV-Infected and HIV-Uninfected Women in Botswana. J Infect Dis.

[11] Dunn DT, Newell ML, Ades AE, Peckham CS (1992). Risk of human immunodeficiency virus type 1 transmission through breastfeeding. The Lancet..

[12] World Health Organization (2013). Consolidated guidelines on the use of antiretroviral drugs for treating and preventing HIV infection: recommendations for a public health approach.

[13] Coovadia HM, Rollins NC, Bland RM, Little K, Coutsoudis A, Bennish ML (2007). Mother-to-child transmission of HIV-1 infection during exclusive breastfeeding in the first 6 months of life: an intervention cohort study. The Lancet.

[14] Wise J (2001). Breast feeding safer than mixed feeding for babies of HIV mothers. BMJ.

[15] UNICEF (2009). Organización Mundial de la Salud. Diarrhoea: why children are still dying and what can be done.

[16] Organization WH, UNICEF (2004). Clinical management of acute diarrhoea.

[17] Gregorio GV, Gonzales MLM, Dans LF, Martinez EG (2016). Polymer-based oral rehydration solution for treating acute watery diarrhoea. Cochrane Database Syst Rev.

[18] Li ST, Grossman DC, Cummings P (2007). Loperamide Therapy for Acute Diarrhea in Children: Systematic Review and Meta-Analysis. PLoS Med.

[19] Allen SJ, Martinez EG, Gregorio GV, Dans LF (2010). Probiotics for treating acute infectious diarrhoea. Cochrane Database Syst Rev.

[20] Bernaola Aponte G, Bada Mancilla CA, Carreazo NY, Rojas Galarza RA (2013). Probiotics for treating persistent diarrhoea in children. Cochrane Database Syst Rev.

[21] Lazzerini M, Ronfani L, The Cochrane Collaboration (2012). Oral zinc for treating diarrhoea in children. Cochrane Database of Systematic Reviews.

[22] Hartling L, Bellemare S, Wiebe N, Russell KF, Klassen TP, Craig WR (2006). Oral versus intravenous rehydration for treating dehydration due to gastroenteritis in children. Cochrane Database Syst Rev.

[23] Aba H, Aminu M (2016). Seroprevalence of hepatitis B virus serological markers among pregnant Nigerian women. Ann Afr Med.

[24] Atia AN, Buchman AL (2009). Oral rehydration solutions in non-cholera diarrhoea: a review. Am J Gastroenterol.

[25] Thom K, Forrest G (2006). Gastrointestinal infections in immunocompromised hosts. Curr Opin Gastroenterol.

[26] Ramos-Soriano AG, Saavedra JM, Wu TC, Livingston RA, Henderson RA, Perman JA (1996). Enteric pathogens associated with gastrointestinal dysfunction in children with HIV infection. Mol Cell Probes.

[27] Guarino A, Bruzzese E, De Marco G, Buccigrossi V (2004). Management of gastrointestinal disorders in children with HIV infection. Paediatr Drugs.

[28] Fontaine O, Kosek M, Bhatnagar S, Boschi-Pinto C, Chan KY, Duggan C (2009). Setting Research Priorities To Reduce Global Mortality from Childhood Diarrhoea by 2015. PLoS Med.

[29] World Health Organization (2010). WHO recommendations on the management of diarrhoea and pneumonia in HIV-infected infants and children.

[30] Motaze NV, Nwachukwu CE, Humphreys EH (2013). Treatment interventions for diarrhoea in HIV-infected and HIV-exposed children. Cochrane Database Syst Rev.

[31] Higgins JPT, Green S Cochrane Handbook for Systematic Reviews of Interventions Version 510 [updated March 2011]. 2011.

[32] Review Manager (Revman) (2011). Copenhagen: the Nordic Cochrane Centre.

[33] Guyatt GH, Oxman AD, Vist GE, Kunz R, Falck-Ytter Y, Alonso-Coello P (2008). GRADE: an emerging consensus on rating quality of evidence and strength of recommendations. BMJ.

[34] Mda S, van Raaij JMA, de Villiers FPR, MacIntyre UE, Kok FJ (2010). Short-term micronutrient suplementation reduces the duration of pneumonia and diarrhoeal episodes in HIV-infected children. J Nutr.

[35] Amadi B, Mwiya M, Musuku J, Watuka A, Sianongo S, Ayoub A (2002). Effect of nitazoxanide on morbidity and mortality in Zambian children with cryptosporidiosis: a randomised controlled trial. The Lancet.

[36] Amadi B (2005). Improved Nutritional Recovery on an Elemental Diet in Zambian Children with Persistent Diarrhoea and Malnutrition. J Trop Pediatr.

[37] Luabeya K-KA, Mpontshane N, Mackay M, Ward H, Elson I, Chhagan M (2007). Zinc or Multiple Micronutrient Supplementation to Reduce Diarrhea and Respiratory Disease in South African Children: A Randomized Controlled Trial. PLoS ONE.

[38] Rollins N, van den Broeck J, Kindra G, Pent M, Kasambira T, Bennish M (2007). The effect of nutritional support on weight gain of HIV-infected children with prolonged diarrhoea. Acta Paediatr.

